# Anorexia Nervosa, Autism, and the ADOS: How Appropriate Is the New Algorithm in Identifying Cases?

**DOI:** 10.3389/fpsyt.2019.00507

**Published:** 2019-07-18

**Authors:** Felicity Sedgewick, Jess Kerr-Gaffney, Jenni Leppanen, Kate Tchanturia

**Affiliations:** Eating Disorders Unit, King’s College London, London, United Kingdom

**Keywords:** anorexia nervosa, autism, comorbidity, women, clinical interview

## Abstract

Thirty years of scholarship has suggested that anorexia nervosa (AN) may be a “female presentation” of autism, supported by work which has found elevated rates of autism traits and diagnoses among women with clinical levels of AN. These traits are often assessed using the Autism Diagnostic Observation Schedule 2nd Edition (ADOS-2), considered the “gold-standard” tool. Recently, the authors of the ADOS-2 revised the diagnostic algorithm for the adult version of the assessment—the one most often used with AN patients. We therefore examined differences in the scores, rates of diagnosis, and correlations with other mental health issues between the two diagnostic algorithms among women with and without AN. One hundred seventy-five women with current AN, who had recovered from AN, and with no history of AN, between the ages of 12 and 53, took part in an ADOS-2 assessment. Their scores were then calculated according to both the original and the new algorithms. The new ADOS-2 algorithm identifies more women as potentially being on the autism spectrum than the old algorithm. Under both algorithms, more currently ill AN patients were identified as potentially being autistic than those with no history of AN. Recovered individuals represented a midpoint between the scores of those with and without AN on both algorithms. There were no correlations with mental health scores in any group, meaning that the new ADOS-2 algorithm is not falsely identifying anxious behaviors or depressive presentations as signs of autism in this group. Overall, we found that more AN patients and recovered individuals scored above cut-off on the new ADOS-2 algorithm, suggesting that women who experience AN may have more autistic traits, which in part persist following weight restoration and recovery. However, the ADOS-2 should not be used alone but in combination with broader clinical assessments to determine whether an autism diagnosis is appropriate for these women.

## Introduction

Autism is a neurodevelopmental condition which is present from early childhood, although it may not be identified until much later, especially in girls and women (1). It is associated with a range of difficulties and differences in the realms of social imagination, social communication, restricted and repetitive behaviors and interests (RRBIs), and sensory processing (2). Autistic individuals have greater cognitive rigidity than neurotypical controls (3, 4), showing a preference for sameness (5). Autistic people have been also shown to have a detail-oriented focus over global-oriented focus when undertaking visual search tasks (6, 7) and more generally (8, 9). Furthermore, difficulties in social relationships, including social imagination and social communication difficulties, are a core diagnostic feature of autism (2).

Anorexia nervosa (AN) is a complex mental health condition characterized by relentless pursuit of weight loss and extreme thinness (2). In addition to eating-related symptoms, theoretical models and empirical data suggest that social–emotional and cognitive difficulties play an important role in the development and progression of AN (10–12). Experimental findings lend support to these models demonstrating that people with AN

show reduced social–emotional communication, greater attentional bias towards negative emotional cues, and poorer theory of mind relative to healthy control (HC) participants (13–16). Furthermore, people with AN have also been found to have greater detail focus at the expense of the big picture, poor cognitive flexibility, and strict rule adherence when compared to HCs (17–19). Together, these findings have led to increasing interest in the theorized association between AN and autism.

An association between autism and AN in women has been theorized since the 1980s (20) and in the decades since there has been a rapid expansion in the amount of research which supports this connection. A recent systematic review found that between 4.0 and 33.0% of people with acute AN scored above the cut-off on self-report questionnaire measures assessing the presence of autistic features (21). The authors also found that studies using clinician or researcher administered diagnostic interview schedules, such as the Autism Diagnostic Observation Schedule 2nd Edition (ADOS-2) (22), found that 4.5–53% of people with acute AN scored above the cut-off for a diagnosis of autism (21). Interestingly, only one of the included studies examined autistic features among people who had recovered from AN and reported that 21% of them scored above diagnostic cut-off (23). This is an interesting finding considering the impact that severe malnutrition can have on social and cognitive functioning, including social withdrawal, difficulties concentrating, and apathy (24). Taken together, there appears to be an over-representation of autism in acute AN, and further examination of presence of autism among people who have recovered from AN would be of interest.

Recently, the authors of the original ADOS have developed a new diagnostic algorithm for Module 4 of the schedule, which is designed to be conducted with individuals who are verbally fluent and for whom it is no longer developmentally appropriate to play with toys and figurines, i.e., older adolescents and adults (22). This follows a full revision of the other modules of the original ADOS-G (25) to create the ADOS-2 (22). The new algorithm was designed to be more sensitive to symptom severity and reflects a move to give more weight to the sensory sensitivities and sensory-motivated behaviors of autistic people, following the inclusion of sensory sensitivities in the diagnostic criteria in the *Diagnostic and Statistical Manual 5th Edition* (DSM-V) (2) definition of autism. However, psychomotor agitation, including tapping, restlessness, and fidgeting, may be easily confused as sensory-motivated behaviors. Therefore, as psychomotor agitation is a common feature of both anxiety and mood disorders (26), careful assessment of usefulness of the new ADOS-2 Module 4 algorithm among highly anxious and depressed individuals, including people with AN, is of interest.

Therefore, in the current study, we sought to explore the use of the new ADOS-2 Module 4 algorithm among women and adolescent girls with current AN, those who have recovered from AN (REC), and those who have never experienced an eating disorder (HC). We expected to see higher levels of autistic traits in patients and recovered individuals than in healthy controls, both under the old and new ADOS-2 algorithm. We also expected that more possible autism cases would be identified by the new algorithm than the old algorithm in all groups. Further to this, we expected that current AN patients would show more autistic behaviors than recovered individuals, as there is a theory that being in a state of malnutrition may exacerbate or mimic autistic traits (24). Finally, we also explored the potential relationships between external presentation of autistic behaviors as measured by the original and new ADOS-2 algorithms and self-reported mental health as well as state of malnutrition as measured by body mass index (BMI) within the AN and REC groups.

## Methods

### Participants

One hundred seventy-five women and girls between the ages of 12 and 53 took part in the study. Sixty-three were HC, 66 were currently ill with AN (AN), and 46 were recovered from AN (REC). The groups were matched in terms of IQ, and each group had a similar ethnic make-up. All AN and REC participants had clinical diagnoses of anorexia nervosa according to the DSM-V (American Psychiatric Association, 2013). Participants were recruited from a range of clinical and community sites across the UK, under ethical approval from the London Surrey Research Ethics Committee (17/LO/2071; 17/LO/1960). All participants gave written informed consent before taking part in the study, and written parental consent was obtained for participants under the age of 16. All procedures were conducted in accordance with the latest version of the Declaration of Helsinki.

### Measures

#### AQ-10

The Autism Quotient-10 item version (27) is a brief 10-item screening questionnaire assessing the presence of autistic features. The questionnaire gives a maximum score of 10, and 6 is generally used as the threshold a potential autism case.

#### EDE-Q

The Eating Disorder Examination Self-Report Questionnaire (28) is a 36-item self-report questionnaire assessing eating disorder psychopathology over the past 28 days.

#### HADS

The Hospital Anxiety and Depression Scale (29) is a 14-item self-report questionnaire assessing levels of both anxiety and depression over the past 2 weeks. Answers are summed for a maximum score of 42. The present study used the total score to assess level of self-reported mood and anxiety symptoms.

#### ADOS-2

The Autism Diagnostic Observation Schedule 2nd Edition (22) is a clinician- or researcher-administered structured observation, generally lasting from 30 min to an hour. It consists of a range of activities and interview questions designed to elicit behaviors and cognitions traditionally associated with autism. These visible behaviors and discussions are then scored from 0 to 3 for “autism severity,” with a score of 3 on any one item reflecting that the person behaved in a highly autistic way, and a score of 0 indicating that the person behaved as would be expected for a neurotypical individual. Under the original algorithm, 11 items from the larger scoring matrix are then summed to create an ADOS score, where 7 is the cut-off for being designated as “on the autism spectrum,” and 10 is the cut-off for being designated as “autistic.” The new algorithm comprises two subscales: social affect (SA) and restrictive and repetitive behaviors (RRBs). For the diagnostic cut-off, the sum of the two subscales is used, with a score of 8 or more indicating an autism diagnosis.

## General Procedure

Participants were all seen at the Institute of Psychiatry, Psychology, and Neuroscience, King’s College London. The data were collected as part of a larger study. Participants completed demographic information, the EDE-Q, and the HADS as part of online questionnaires. The larger testing session lasted ∼3 h, including the ADOS-2, a range of neurocognitive tests, and a structural and functional MRI scan (reported elsewhere). The ADOS-2 was scored immediately after the testing session by the administering researcher, and 20% of videos were double coded by other ADOS-2-trained researchers within the group, resulting in an inter-rater reliability of 87%. Two videos were further included in a wider reliability meeting with members of other research groups within the institution, and the inter-rater reliability for these was 81%, with group consensus codes being used in the final analysis.

## Data Analysis

All data analyses were conducted with R (30). Due to differences in group sizes, the presence of significant heteroscedasticity was assessed using the Breusch–Pagan test (31–33). Where significant heteroscedasticity was present, group differences in clinical and demographic measures as well as in ADOS-2 scores were assessed using robust M-estimator with Huber’s approach for controlling for influential outliers (34). In the absence of significant heteroscedasticity, group differences were investigated with multiple linear regressions. Significant differences in the number of individuals who met the criteria for autism spectrum diagnosis under the original and new algorithms were examined using generalized linear binomial mixed model (35). Significance level was set at *p* < 0.05.

Within the AN and REC groups, Spearman’s correlations between ADOS-2 scores under the original and new algorithms and self-reported clinical variables including eating disorder symptomatology, depression, and anxiety, as well as BMI were explored. Correlations analyses were corrected for multiple comparisons using false discovery rate set at *q* = 0.05, and *p* < 0.009 was considered significant (36).

## Results

### Clinical and Demographic Characteristics

The sample characteristics are presented in **Table 1**. As expected, there was a significant group difference in BMI, with AN participants having a significantly lower BMI than both HC and REC participants [AN vs. HC: *t*(172) = −10.73, *p* < 0.001; AN vs. REC: *t*(172) = −7.56, *p* < 0.001; HC vs. REC: *t*(172) = 2.12, *p* = 0.089]. There was also a significant difference in age between the groups such that the REC group was significantly older than the HC [*t*(172) = −3.07, *p* = 0.007] and the AN group [*t*(172) = −3.51, *p* = 0.002]. There was no significant difference in age between the AN and HC participants [*t*(172) = −0.45, *p* = 0.896]. Finally, there was significant heteroscedasticity in the HADS (BP = 14.78, *p* < 0.001), EDE-Q (BP = 10.05, *p* = 0.007), and AQ-10 (BP = 9.38, *p* = 0.009) total scores between the groups, and group differences were examined with a robust M-estimator. As expected, the AN group reported significantly more eating disorder symptomatology than the HC (*z* = 17.21, *p* < 0.001) and REC groups (*z* = 13.28, *p* < 0.001). Interestingly, the REC group also reported significantly more eating disorder symptomatology than the HC group (*z* = −2.59, *p* = 0.026). Both AN and REC groups also reported significantly more depression and anxiety than the HC participants, with the currently ill AN group reporting more mood and anxiety symptoms than those who were recovered (AN vs. HC: *z* = 13.18, *p* < 0.001; AN vs. REC: *z* = 6.68, *p* < 0.001; HC vs. REC: *z* = –5.50, *p* < 0.001). Finally, the AN and REC groups also reported more autistic features than the HC group (AN vs. HC: *z* = 5.07, *p* < 0.001; AN vs. REC: *z* = 1.69, *p* = 0.210; HC vs. REC: *z* = −2.93, *p* = 0.009).

**Table 1:** Demographic and clinical information about participants by group.

Measure	HC Mean (SD)	AN Mean (SD)	REC Mean (SD)	*F* statistic, *p* value
Age	21.48 (3.95)	21.14 (5.64)	24.95 (7.43)	*F*(2) = 6.94, *p* = 0.001
IQ	110.10 (5.56)	108.16 (5.43)	110.30 (5.70)	*F*(2) = 1.35, *p* = 0.27
BMI	21.76 (2.15)	17.22 (2.56)	21.01 (2.40)	*F*(2) = 60.87, *p* < 0.001
EDE-Q total	0.82 (0.95)	3.85 (1.37)	1.54 (1.59)	*F*(2) = 86.14, *p* < 0.001
HADS total	7.10 (4.12)	22.42 (7.89)	13.79 (6.90)	*F*(2) = 75.14, *p* < 0.001
AQ-10	2.07 (1.49)	3.98 (2.38)	3.42 (2.46)	*F*(2) = 12.99, *p* < 0.001

N.B. IQ, intelligence quotient; BMI, body mass index; EDE-Q, Eating disorders examination questionnaire; HADS, hospital anxiety and depression scale; AQ-10, autism quotient (short version); HC, healthy control; AN, anorexia nervosa; REC, recovered from anorexia nervosa; SD, standard deviation.

### ADOS Scores

#### Original Algorithm

**Figure 1:**
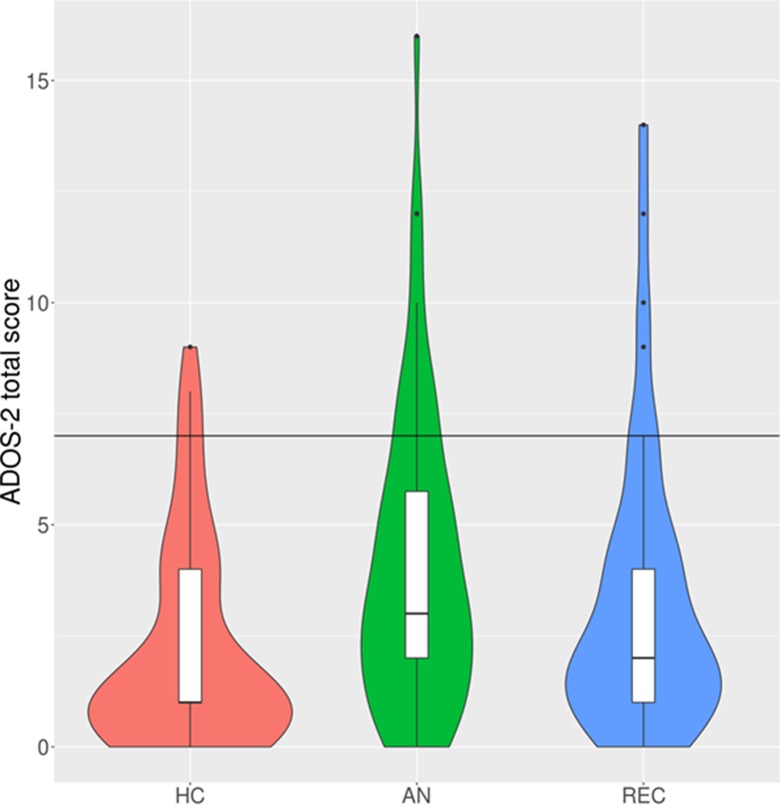
Violin and box plots of Autism Diagnostic Observation Schedule 2nd Edition (ADOS-2) total scores under the original algorithm. N.B. The black horizontal line indicates the cut-off for autism diagnosis (total score of 7 or higher). HC, healthy control; AN, anorexia nervosa; REC, recovered from anorexia nervosa. The violin plots demonstrate the density of the scores within each group. The box plots show the median, interquartile range, minimum, and maximum scores within each group.

The multiple linear regression showed that there was a significant effect of group on ADOS-2 score (**Table 2**; **Figure 1**). *Post hoc* *t*-tests revealed that this was driven by a significant difference between AN patients and HC participants, such that currently ill AN patients scored significantly higher on the old ADOS-2 algorithm, *t*(172) = 3.68, *p* < 0.001. There was no significant difference in ADOS-2 scores between the AN and REC groups, *t*(172) = 1.37, *p* = 0.359, or the REC and HC groups, *t*(172) = −1.99, *p* = 0.118.

**Table 2:** Autism Diagnostic Observation Schedule 2nd Edition (ADOS-2) scores on the original and new algorithms by group.

Measure	HC Mean (SD)	AN Mean (SD)	REC Mean (SD)	*F* statistic, *p* value
Original algorithm	2.14 (2.35)	4.06 (3.30)	3.28 (3.19)	*F*(2) = 6.82, *p* = 0.001
New algorithm	3.21 (2.47)	5.65 (3.81)	4.74 (4.06)	*F*(2) = 7.97, *p* < 0.001

N.B. HC, healthy control; AN, anorexia nervosa; REC, recovered from anorexia nervosa; SD, standard deviation.

#### New Algorithm

**Figure 2:**
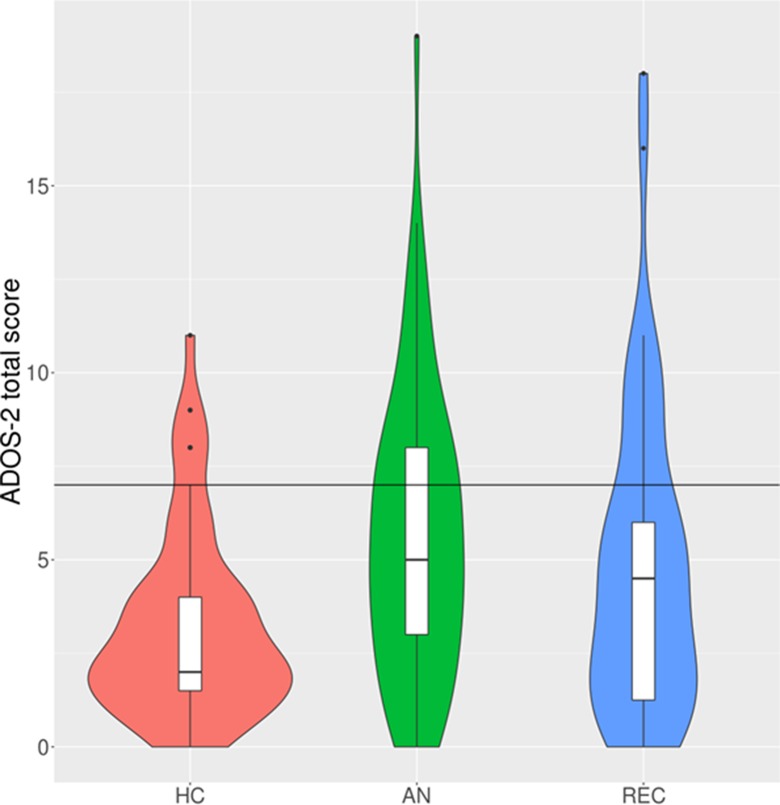
Violin and box plots of ADOS-2 total scores under the new algorithm. N.B. The black horizontal line indicates the cut-off for autism diagnosis (total score of 7 or higher). HC, healthy control; AN, anorexia nervosa; REC, recovered from anorexia nervosa. The violin plots demonstrate the density of the scores within each group. The box plots show the median, interquartile range, minimum, and maximum scores within each group.

Due to significant heteroscedasticity, group differences in ADOS-2 scores calculated based on the new algorithm were investigated through robust M-estimator. There was a significant difference between the groups (**Table 2**; **Figure 2**), and *post hoc* *z*-tests revealed that this significance arose from a significant difference between HC participants and the AN patients (*z* = −4.34, *p* < 0.00) and REC patients (*z* = −2.56, *p* = 0.028). There was no significant difference in the new ADOS-2 scores between the AN and REC groups (*z* = 0.1.92, *p* = 0.134) or HC and REC groups (*z* = −1.72, *p* = 0.199).

#### Comparing the Original and New Algorithms

**Figure 3:**
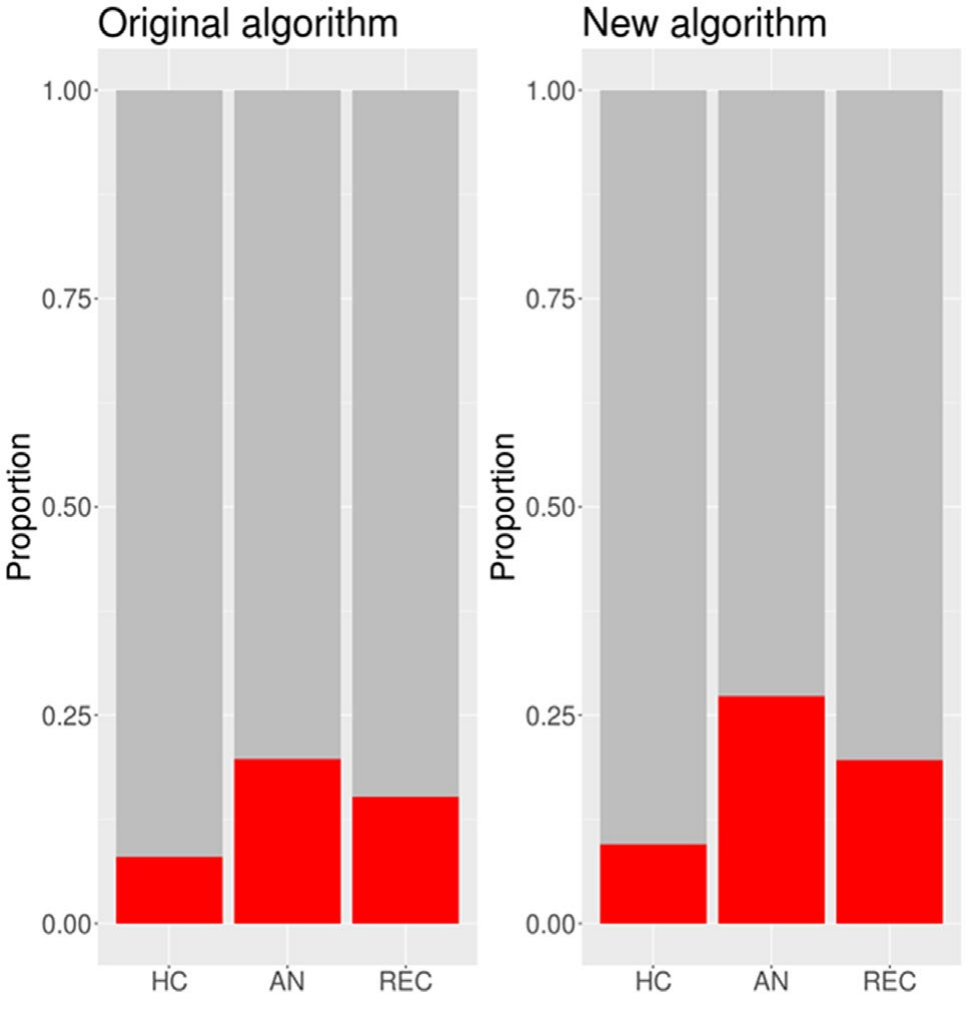
Proportions of participants in each group scoring above the cut-off under the original and new algorithms. N.B. HC, healthy control; AN, anorexia nervosa; REC, recovered from anorexia nervosa.

The proportion of participants in each group scoring above the cut-off under the original and new algorithms is presented in **Figure 3**. A generalized binomial linear mixed effects model showed that there was a significant difference in the number of people meeting cut-off for autism spectrum diagnosis between the original and new algorithms (*b* = −5.18, SE = 2.57, *z* = −2.02, *p* = 0.044). Under the original algorithm, five HC participants (7.9%), 13 AN participants (19.7%), and 7 REC participants (15.2%) scored above the cut-off of 7. Under the new algorithm, six HC participants (9.5%), 18 AN participants (27.3%), and 9 REC participants (19.6%) scored above the cut-off of 8.

#### ADOS and Clinical Characteristics

Correlations between clinical characteristics and autistic symptomatology assessed with ADOS-2 original and new algorithms can be seen in **Table 3**. There was a significant correlation between HADS total score and ADOS-2 scores and between AQ-10 and ADOS-2 scores within the REC group only under the original algorithm. There were no other correlations between the measures within the AN or the REC group that met the corrected *p* < 0.009 threshold for significance.

**Table 3:** Correlations between clinical characteristics and ADOS-2 original and new scores in the AN and REC groups.

Measure	Original algorithm		New algorithm	
	AN	REC	AN	REC
BMI	ρ = −0.25, *p* = 0.044	ρ = −0.05, *p* = 0.748	ρ = −0.15, *p* = 0.217	ρ = 0.03, *p* = 0.818
EDE-Q total	ρ = 0.10, *p* = 0.453	ρ = 0.30, *p* = 0.046	ρ = 0.11, *p* = 0.420	ρ = 0.26, *p* = 0.089
HADS total	ρ = 0.11, *p* = 0.415	ρ = 0.44, *p* = 0.0027	ρ = 0.08, *p* = 0.552	ρ = 0.32, *p* = 0.032
AQ-10	ρ = 0.10, *p* = 0.461	ρ = 0.42, *p* = 0.005	ρ = 0.13, *p* = 0.318	ρ = 0.34, *p* = 0.026

N.B. HC, healthy control; AN, anorexia nervosa; REC, recovered from anorexia nervosa; BMI, body mass index; EDE-Q, Eating disorders examination questionnaire; HADS, Hospital anxiety and depression scale; AQ-10, Autism Quotient (short version).

## Discussion

The aim of the present study was to investigate the usefulness of the new ADOS-2 Module 4 algorithm among a highly anxious and depressed group of people with AN as well as those who had recovered from AN and people with no history of eating disorders. Importantly, the new ADOS-2 algorithm identifies significantly more currently ill and recovered AN individuals as being on the autism spectrum. The scores from the new algorithm did not correlate significantly with self-reported depression, anxiety, or eating disorder symptomatology. This suggests that the new ADOS-2 algorithm may be useful in identifying AN patients who have co-occurring autism and who may therefore benefit from adapted treatment approaches which account for their possible cognitive and neurodevelopmental differences (37, 38).

As hypothesized, there were significantly more AN participants who met cut-off for autism diagnosis than HC or recovered participants on both the new and the old ADOS-2 algorithms. This echoes previous literature that has documented an over-representation of autistic features among those with AN compared to those without AN (21). Although there were no significant differences between currently ill AN and recovered participants on their ADOS-2 scores, inspection of the median scores per group suggested that recovered participants may represent a midpoint between currently ill AN and HC participants. The present finding could be taken to suggest that rather than having underlying autism, the fact that acutely ill AN patients present with more autistic behaviors may be related to state of illness. However, this is in direct contrast with previous findings showing that similar proportions of participants in currently ill AN and recovered groups scored above the cut-off on the ADOS-2 (23). Therefore, further longitudinal work is needed to investigate the stability of autistic features through recovery among people with AN.

This interpretation is bolstered by the lack of significant correlations of new algorithm ADOS-2 scores with EDE-Q and BMI. This supports the idea that the new ADOS-2 algorithm is not picking up behaviors associated with anorexia nervosa rather than an underlying autism diagnosis and therefore is not overly sensitive in this population resulting in false-positive autism diagnoses. Furthermore, the fact that the scores on the new ADOS-2 algorithm did not correlate with self-reported depression or anxiety suggests that this algorithm does not erroneously pick up behaviors that may be associated with psychomotor agitation. These findings speak in favor of using the ADOS-2 to assess the presence of autistic features not only among people with eating disorders but also more widely among people with mood and anxiety disorders.

### Limitations

While this paper highlights the usefulness of the new ADOS-2 algorithm among those with AN, it also had some limitations. First, it is important to recognize that regardless of algorithm used, the ADOS-2 alone is not enough to confidently assume that underlying autism is present in AN patients. While the administration of an ADOS-2 with AN patients who may be autistic is an important first step, a full clinical assessment, with elements such as a developmental history, is essential before giving an autism diagnosis. However, if a patient’s ADOS-2 scores suggest that autism may be present, it is important to build this into treatment plans as early as possible while waiting for an autism diagnosis appointment, which can take several months.

Second, although the present findings suggest that recovered individuals may represent a midpoint between currently ill AN and HC participants on the ADOS-2 scores, it is important to note that the present study was cross-sectional. Therefore, longitudinal work is needed to further explore the possibility that the presence of autistic features in AN as measured with the ADOS-2 may be related to illness state rather than a reflection of underlying autism. Recognizing the impact of illness state on ADOS-2 score is crucial, as autism is a lifelong diagnosis, and a false diagnosis could negatively affect ED treatment, as many clinicians report having low confidence in their ability to work with an autistic person (39).

Third, the present study assessed the presence of eating disorder symptomatology, mood, and anxiety symptoms using self-report rather than clinical assessments. To confirm that the ADOS-2, in general, and the new algorithm, in particular, do not erroneously pick up psychomotor agitation or other behavior symptoms related to mental health as unusual sensory interest, replication of the present findings with clinician assessment of mood and anxiety is key. Mislabeling of psychomotor symptoms as autistic features could generate false positive diagnoses that may have a negative impact on illness outcome or treatment. Therefore, further research into the specific nature of these behaviors in those with AN and potential autism is needed.

Fourth, the present study did not have a comparison group of girls and women with a clinician-confirmed autism diagnosis, who would have given a sense of the changes in ADOS-2 scores generated using the new algorithm with participants who have established autism, and whether the differences in our sample are seen more generally. Furthermore, we did not have a subgroup of participants with AN and clinician-confirmed autism. Inclusion of such a group would be necessary to fully investigate the sensitivity of the new ADOS-2 algorithm in detecting autism within this patient population. Therefore, further research assessing the sensitivity of both the original and new ADOS-2 algorithms under double-blind conditions with autistic and neurotypical participants with and without AN is needed.

Finally, the groups were not exactly matched in size, and significant heteroscedasticity was present in some of the measures. Although steps were taken to conduct robust statistical tests to avoid false findings arising from the presence of heteroscedasticity, future work would benefit from having larger samples sizes and using purposeful matched sampling, especially for recovered individuals.

## Conclusions

In conclusion, our findings suggest that the new ADOS-2 algorithm is a useful measure of autistic traits among women with current AN and who have recovered from AN. Our findings suggest that some autistic traits remain after recovery, which may reflect a genuine underlying autism diagnosis. However, the present study is cross-sectional, and the findings need to be confirmed with a longitudinal study of autistic traits before any firm conclusions can be drawn.

## Ethics Statement

Participants were recruited from a range of clinical and community sites across the UK, under ethical approval from the London Surrey Research Ethics Committee (17/LO/2071; 17/LO/1960). All participants gave written informed consent before taking part in the study and all procedures were conducted in accordance with the latest version of the Declaration of Helsinki.

## Author Contributions

FS carried out recruitment, testing, analysis, and write-up for this research and manuscript. JK-G contributed data and to the write-up of this manuscript. JL carried out recruitment, analysis, and write-up for this research and manuscript. KT led the research group within which this work was conducted and was awarded the funding which enabled it to take place, as well as proofreading the manuscript.

## Conflict of Interest Statement

The authors declare that the research was conducted in the absence of any commercial or financial relationships that could be construed as a potential conflict of interest.
